# Health insurance in private and public health facilities in Southwestern Nigeria: what determines clients´ satisfaction with quality of service?

**DOI:** 10.11604/pamj.2022.41.268.26875

**Published:** 2022-04-01

**Authors:** Roseline Oluyemisi Akande, Olugbemiga Lanre Abodunrin, Sunday Olakunle Olarewaju, Adeleye Abiodun Adeomi, Joel Olufunminiyi Akande, Ifedola Olabisi Faramade

**Affiliations:** 1 Department of Community Medicine, Bowen University, Iwo, Osun State, Nigeria,; 2 Department of Community Medicine, LAUTECH Teaching Hospital, Ogbomoso, Oyo State, Nigeria,; 3 Department of Community Medicine, Osun State University, Osogbo, Osun State, Nigeria,; 4 Department of Community Health, Obafemi Awolowo University, Ile-Ife, Osun State, Nigeria,; 5 Department of Chemical Pathology, Bowen University Iwo, Osun State, Nigeria,; 6 Department of Community Medicine, LAUTECH Teaching Hospital, Osogbo, Osun State, Nigeria

**Keywords:** Clients, satisfaction, private, public, health facilities, health insurance

## Abstract

Introduction: insured-persons have complained about poor quality of services rendered by health care providers, which has consequently affected their satisfaction with care received. The objectives of this study aimed to identify the determinants of satisfaction and compare the level of clients´ satisfaction with quality of care received in both public and private health care facilities, in Oyo-State, Nigeria.

Methods: this was a cross sectional study, comparative in design. A total number of 300 clients were recruited from selected public and private health facilities in Oyo-state, using a multistage sampling technique. Data were analyzed using IBM SPSS version 24, and the level of significance was set at p-value < 0.05.

Results: the mean age of the respondents in private and public health facilities was 39.9 ± 10.0 years and 42.4 ± 10.1 years respectively. About 74% and 41.3% of the enrolees in the public and private health facilities respectively were dissatisfied with waiting time before receiving care with a statically significant difference of p=0.002. Majority of the respondents (82.7%) in the public health facilities and only 42.7% of those using private health care facilities were satisfied with the quality of drugs given to them at their respective pharmacies. This finding was statistically significantly different with p=0.001. Overall level of satisfaction with quality of care was 60% and 40% among enrolees using public and private health facilities respectively. There was a statistically significance difference (p=0.028) between the overall level of satisfaction and the type of health facility used by the clients. The determinants of clients´ satisfaction with quality of care in both private and public health facilities in this study were mainly socio-demographic characteristics; age (p=0.007), level of education (p=0.046) and occupation (p=0.004), the waiting time experience and the type of facility where services were accessed.

Conclusion: clients attending public health facilities were more satisfied with care received under NHIS, compared with those using private health facilities. Efforts should be made to reduce waiting time and improve quality of drugs in the public and private facilities respectively.

## Introduction

Health insurance in developing countries provide immense opportunities for people, especially the poor to access healthcare anytime the need arises [1]. It therefore serves as a means of promoting universal health coverage (UHC) as well as achieving the sustainable development goal 3 which seek to ensure healthy lives and promote well-being for everyone at all ages [1-3]. Despite this indisputable fact, Nigeria like most African countries, has less than 5% of its populace having health insurance coverage, with majority of the insured clients working in the formal sector and a dismal coverage observed in the informal sector [4]. Furthermore, reports of satisfaction surveys on quality of care done across Nigeria among National Health Insurance scheme (NHIS) enrollees has corroborated clients satisfaction as an important outcome of health services performances [3,5,6]. Generally, satisfaction of patients is hinged on and often derived from their perceptions about the conduciveness of the facility´s environment before and following a clinic visit [7]. It is also associated with the quality of health care provided at the health facility, determined by the health care provider´s expertise, confidentiality, promptness in emergency care, short waiting time, accuracy of laboratory investigations and quick pharmacy services [3,8].

The NHIS also offers clients´ the right to choose where to access health care either with public or private accredited health providers once they are enrolled under the scheme [9]. Subsequently, enrollees often choose their healthcare providers on the basis of the satisfaction they received from a particular healthcare facility and their perceived quality of care [9]. The possibility of switching to another healthcare provider by clients enrolled under NHIS who are dissatisfied with care received have been reported by previous studies and can be a key indicator for the need for quality improvement in service delivery [1,8,10,11]. Satisfaction surveys across Nigeria, showed lesser satisfaction with quality of care from patients whose views were sought in public tertiary institutions [11,12]. It was also observed in the different geo-political zones in Nigeria, that the poor performance of public providers was attributable to giving patients an appointment for a particular day without a specific patient consultation time and increased numbers of patient exceeding the capacity of the public health facilities. In addition, poor quality of public health services resulting from bureaucracy in registration processes at the health records department, poor attitude of providers to the insured-users and bad interpersonal relationship also contributed to enrollees´ dissatisfaction [5,10]. Clients that are satisfied with the quality of care are more likely to seek medical consultation in the hospital, stick to treatment plan, attract new enrollees within the community to the facility, make a more informed choices about the health care providers and encourage a continuous quality improvement in the hospital compared with dissatisfied clients [12]. Although the scheme has been in operation for several years, and many studies have assessed satisfaction of enrollees with the quality of care provided under the scheme, few researches however exist on comparison between level of clients´ satisfaction with quality of health care services received by enrollees under NHIS in public and private health care services. Understanding the views of clients on the quality of health care and comparing these views across accredited private and public health facilities will offer policy makers and health managers, the opportunity to address existing gaps in the service delivery process and promote client trust in the healthcare system and the National health insurance scheme. Objectives: i) to identify the determinants of satisfaction with quality of care received in both public and private health care facilities, in Oyo-State, Nigeria, ii) to compare the level of clients´ satisfaction with quality of care received in both public and private health care facilities, in Oyo-State, Nigeria.

## Methods

**Study setting**: the study was carried out in Oyo State. Oyo-state, an inland state in the South-western Nigeria, with Ibadan being the State capital. The state consists of thirty- three local government areas which are broadly divided into twelve urban, nine semi-urban and twelve rural Local Governments. Oyo-State has a total number of two-hundred and two (202) NHIS accredited Health Care Providers (HCPs) which provides primary, secondary and tertiary level of care for the enrollees under the scheme [13]. Justification: for the sustainability of the NHIS in Nigeria, continuous feedback from the clients´ perspective are needed for the improvement in the quality of health services rendered. Therefore, this study seeks to understand the concerns of public and private health facilities NHIS enrollees about the quality of care received, thereby improving health services delivery and healthcare satisfaction within the Nigerian health system.

**Study design and population**: this was a cross sectional study, comparative in design with the study population being NHIS registered clients receiving care from accredited public and private health facilities in Oyo-State. Inclusion criteria were clients registered with NHIS under private and public health facilities and who had been receiving care for at least one year.

**Sample size determination** : the sample size was calculated using theformula for comparing two groups:


$$N=\frac{2{\left(Z\alpha +Z\beta \right)}^{2}p_{0}(1-p_{0})}{d^{2}}[14].$$

A non-response rate of 10% was envisaged among the respondents and adjustment for this was made to arrive at a minimum sample size of 150 respondents each in the public and private health care facilities (a total of 300 respondents). The respondents were selected using a multi-stage sampling technique.

**Sampling technique**: the sampling procedure was a three-level multi-stage cluster sampling aimed at selecting eligible persons. Stage one: from the list of the Local Government Areas (LGAs) in two (2) senatorial districts, three urban local government areas were chosen from each of them, using simple random sampling (balloting method), giving a total of six (6) LGAs. Stage two: from the list of all accredited NHIS health care providers (both public and private) in the six selected LGAs, two public and two private health facilities were selected by simple random sampling via balloting method, making a total of twenty-four facilities (Twelve facilities each for both the public and private facilities). Stage three: systematic random sampling technique was then used to select eligible respondents from the twenty-four selected health facilities. Respondents were selected by systematic sampling, (with the sampling interval determined based on the number of NHIS clients attending the health facilities daily and the number of respondents to be selected each day from the patient daily list). The first respondent in each facility used, was selected by simple random sampling through balloting method and subsequent respondent (Kth respondent) was selected by using the sampling interval obtained from the patient daily list.

**Research instrument and data collection methods**: data was collected using an interviewer-administered semi-structured questionnaire. The questionnaire contained questions adapted from standard modified patients satisfaction questionnaires [15-17] and self-developed questions.

**Validation and pretest of the instrument**: the validity and reliability of the questionnaire were done before the final collection of data. Three Nigerian experts in the field of epidemiology and medical statistics in a Nigerian university evaluated the extent to which the variables in the questionnaires were relevant to the objectives of the study. Thereafter, the questionnaire was pretested among NHIS registered clients that access health services in a public and private health care facility in Osun-State. These selected respondents had similarities with those who were recruited for the main study and shared similar socio-demographic and socio-economic characteristics. The pretest helped to assess the relevance of the questions in producing responses from the participants. Ambiguous questions were either removed or rephrased in line with study objectives. Cronbach´s alpha internal consistency reliability of 0.86 was achieved for the analyzed variables.

**Measurement of main outcome variables**: the primary outcome measure of the study was satisfaction. Questions were asked about the satisfaction of respondents with the quality of care received under the scheme. These were measured using the five dimensions of SERVQUAL analysis (empathy, assurance, reliability, responsiveness and tangibility) influencing the clients perception of quality of care [18]. The responses were assessed using five items on a five-point Likert scale ranging from very dissatisfied (1), dissatisfied (2), indifferent (3), satisfied (4), to very satisfied (5). Thereafter, the responses were coalesce into three categories; satisfied, indifferent and dissatisfied. The sums of the scores for individual respondent were calculated, and the mean of all the scores were determined. The mean satisfaction score was 88.8, while the minimum and maximum satisfaction score was 48.0 and 115.0 respectively. The respondents who scored up to or above the mean were categorized as satisfied while those who scored below the mean were categorized as dissatisfied.

**Data analysis**: the questionnaires were sorted out, entered into a computer and the obtained data was analyzed using IBM Statistical Product and Service Solutions (SPSS) version 24. Data were presented using frequency tables and chart. Bivariate analysis was used to determine the relationship between the dependent variables (which includes the satisfaction with the quality of care received by the respondents and factors associated with their satisfaction) and the independent variables (such as age, gender, and ethnicity, level of education, occupation, marital status and religion of the respondents). The categorical variables were assessed using the Pearson Chi-square test. In the multivariate analysis, determinants of satisfaction were ascertained using the logistic regression model. Only variables whose p-values were statistically significant, were entered into the model. The estimated coefficients were expressed as odds ratios (ORs) and their 95% confidence intervals were also calculated. The level of significance for the study was set at p < 0.05.

**Ethical issues**: ethical approval was obtained from the Ethics and Research committee of LAUTECH Teaching Hospital, Ogbomoso, prior to commencement of the study (LTH/OGB/EC/2015/082). Approval to conduct the study was also obtained from the Medical Directors of the health facilities used. The respondents were duly informed about the nature of the study and that participation was voluntary. Individual written informed consent was obtained and information gathered from all the respondents were kept confidential, with identification of the participants using only serial numbers.

## Results

The mean age of the respondents in private and public health facilities was 39.9 ± 10.0 years and 42.4 ± 10.1 years respectively. Expectedly, majority of the clients in both the private and public health facilities had tertiary level of education and were married (Table 1).

**Table 1:** socio-demographic characteristics of respondents by their health facility

Variable	Type of health facility (%)		Statistics
	Private (n = 150)	Public (n = 150)	
**Age groups (in years)**			
21- 40	91 (60.7)	71 (47.3)	χ^2^=5.563
41-60	55(36.7)	72(48.0)	df=2
>60	4(2.7)	7(4.7)	p=0.062
Mean age	39.9 ± 10.0	42.4 ± 10.1	
**Gender**			χ^2^=4.332
Male	80 (53.3)	62(41.3)	df=1
Female	70 (46.7)	88 (58.7)	*p=0.037
**Ethnicity**			
Yoruba	134(89.3)	139(92.7)	+χ^2^=1.892
Hausa	6(4.0)	6(4.0)	df=3
Igbo	7(4.7)	3(2.0)	p=0.585
Others	3(2.0)	2(1.3)	
**Level of education**			
Primary	0	7(4.7)	+χ^2^=10.97
Secondary	20(13.8)	18(12.0)	df=3
Tertiary	121(80.7)	120(80.0)	*p=0.012
Others	9(3.0)	5(3.3)	
**Marital status**			
Single	18(12.0)	4(2.7)	+χ^2^=11.51
Married	128(85.3)	141(94.0)	df=3
Widowed	3(2.0)	2(1.3)	*p=0.009
Separated	1(0.7)	3(2.0)	
**Occupation**			
Civil servants	104(69.3)	114(76.0)	χ^2^=13.132
Private sector employee	29(19.3)	12(8.0)	df=3
Artisan	9(6.0)	5(3.3)	*p=0.004
Unskilled workers	8(5.3)	19(12.7)	
**Religion**			
Christianity	124(82.7)	110(73.3)	+χ^2^=4.842
Islam	26(17.3)	39(26.0)	df=2
Others	0	1(0.7)	p=0.089

* Statistically significant+Likelihood ratio

**Satisfaction with Doctors´ care**: at bivariate level, the summary care of doctors for respondents assessed using the variables; prompt attention (p = 0.055), empathy (p=0.506), physical examination (p=0.376) and preventive health care and lifestyle counseling (p=0.374) were not significantly different between the private and public health facilities enrollees (Table 2).

**Table 2:** satisfaction of NHIS clients with doctors´ care in public and private health facilities

Satisfaction with doctors´ attention	Type of health facility (%)			Statistics
	Private (n=150)	Public ( n=150)	Total (N = 300)	
**Waiting time before receiving doctors’ care**				
Satisfied	45 (30.0)	31 (20.7)	76 (25.3)	χ^2^=12.473
Indifferent	11 (7.3)	6 (4.0)	17 (5.7)	df=2
Dissatisfied	94 (62.7)	113(75.3)	207(69.0)	*p=0.002
**Empathy from the doctor**				
Satisfied	133 (88.7)	137 (91.3)	270 (90.0)	χ^2^=1.361
Indifferent	13 (8.7)	8 (5.3)	21 (7.0)	df=2
Dissatisfied	4(2.7)	5(3.3)	9(3.0)	p =0.506
**Doctor listened to my problems**				
Satisfied	141 (94.0)	146 (97.3)	287 (95.7)	χ^2^=2.020
Indifferent	7 (4.7)	3 (2.0)	10 (3.3)	df=2
Dissatisfied	2 (1.3)	1 (0.7)	3(1.0)	p=0.364
**Doctor physically examined me**				
Satisfied	135 (90.0)	134 (89.3)	269 (89.7)	χ^2^=2.002
Indifferent	5(3.3)	2(1.3)	7(2.3)	df=2
Dissatisfied	10(6.7)	14(9.3)	24 (8.0)	p=0.376
**Doctor gave me information about my illness**				
Satisfied	132 (88.0)	125 (83.3)	257 (85.7)	χ^2^=1.880
Indifferent	7 (4.7)	7 (4.7)	14(4.7)	df=2
Dissatisfied	11(7.3)	18(12.0)	29(9.7)	p=0.391
**Preventive healthcare and life> counseling was given to me**				
Satisfied	125 (83.3)	130 (83.3)	255 (85.0)	χ^2^=1.964
Indifferent	6 (4.0)	8 (5.3)	14(4.7)	df=2
Dissatisfied	19 (12.7)	12(8.0)	31(10.3)	p=0.374

* Statistically significant

**Figure 1:**
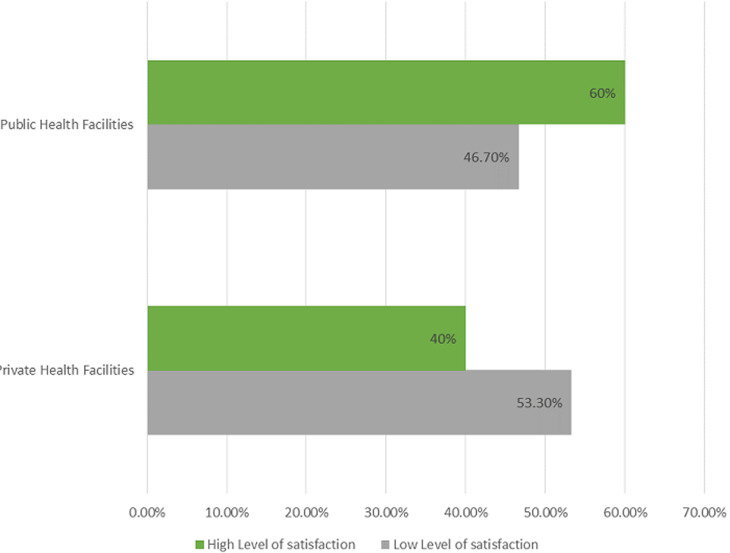
overall satisfaction of NHIS clients in private and public health facilities

**Satisfaction with other clinical services**: a statistically significant association was found between the quality of drugs provided at the pharmacy (p=0.019) among the public and private health facilities respondents while other variables were not statistically significant (Table 3). More than half of the respondents receiving care from private health facilities (53.3%) reported lower satisfaction level compared to their counterparts in the public health facilities (46.7%) while clients in the public health facilities (60.0%) had a higher level of satisfaction compared to those attending private health facilities (40.0%) (Figure 1).

**Table 3:** satisfaction of NHIS clients with clinical services in public and private health facilities

Variable	Type of health facility (%)		Statistics
	Private (n=150)	Public (n=150)	
**Satisfaction with the quality of drugs provided at the pharmacy**			
Agree	94 (62.7)	113(75.3)	χ^2^=7.955
Indifferent	9(6.0)	2(1.3)	df=2
Disagree	47 (31.3)	35 (23.3)	*p=0.019
**I got the services of required specialists under the scheme**			
Yes	96 (64.0)	94 (62.7)	χ^2^=0.078
No	20 (13.3)	20 (13.3)	df=2
Not applicable	34(22.7)	36(24.0)	p=0.962
**Satisfaction with services provided in the lab**			
Yes	104 (69.3)	110(73.3)	χ^2^=0.587
No	16(10.7)	14 (9.3)	df=2
Not applicable	30 (20.0)	26 (17.3)	p=0.746
**I received pleasant treatment from the nursing officers**			
Agree	128 (85.3)	129 (86.0)	χ^2^=0.233
Indifferent	4 (2.7)	5 (3.3)	df=2
Disagree	18 (12.0)	16 (10.7)	p=0.890
**Medical staff correctly diagnose my illness leading to recovery**			
Agree	129(86.0)	135(90.0)	χ^2^=2.936
Indifferent	2(1.3)	4(2.7)	df=2
Disagree	19(12.7)	11 (7.3)	p=0.230
**I feel satisfied with the medical services I received today**			
Agree	117 (78.0)	128 (85.3)	χ^2^=4.075
Indifferent	9 (6.0)	3(2.0)	df=2
Disagree	24(16.0)	19(12.7)	p=0.130
**I will recommend my health care providers to other NHIS enrollees**			
Agree	122(82.4)	124(82.7)	χ^2^=2.030
Indifferent	7 (4.7)	5 (3.3)	df=2
Disagree	19 (12.8)	21 (14.0)	p=0.371

* Statistically significant

**Factors associated with clients´ satisfaction as regards the quality of care received in private and public health facilities**: the factors showing statistically significant association includes socio-demographic characteristics such as age (p=0.007), level of education (p=0.046) and occupation (p=0.004), perceived quality of drugs given to clients at the pharmacy (p=0.001) as well as satisfaction with waiting time (p=0.000) and the health facility type used by enrollees (p=0.028) (Table 4).

**Table 4:** factors associated with clients satisfaction among enrollees in public and private health facilities

Variables	Level of clients satisfaction		Statistics
	Low (%)	High (%)	
**Age groups (in years)**			
21 – 40	88 (55.0)	72 (45.0)	χ^2^=10.883
41 – 60	47 (37.0)	80 (63.0)	df=2
> 60	5 (38.5)	8 (61.5)	*p=0.007
**Gender**			χ^2^=0.569
Male	71 (49.3)	73 (50.7)	df=1
Female	69 (44.2)	87 (55.8)	p=0.451
**Ethnicity**			
Yoruba	127 (46.9)	144 (53.1)	+χ^2^=3.498
Hausa	6 (50.0)	6 (50.0)	df=3
Igbo	4 (33.3)	8 (66.7)	p=0.321
**Level of education**			
Primary	4 (44.4)	5 (55.6)	χ^2^=8.018
Secondary	11 (28.9)	27 (71.1)	df=3
Tertiary	116 (48.5)	123 (51.5)	*p=0.046
Others	9 (64.3)	5 (35.7)	
**Marital status**			
Single	9 (40.9)	13 (59.1)	χ^2^=4.063
Married	122 (45.7)	145 (54.3)	df=3
Widowed	4 (80.0)	2 (20.0)	p=0.255
Separated	3 (75.0)	2 (25.0)	
**Occupation**			
Civil servants	114 (52.3)	104 (47.7)	+χ^2^=13.132
Private sector employee	18 (43.9)	23 (56.1)	df=3
Artisan	2(14.3)	12 (85.7)	*p=0.004
Unskilled workers	6 (22.2)	21 (77.8)	
**Long waiting time at the facilities (> 1 hour)**			χ2=4.908
Private	62 (41.3)	88 (58.7)	df=1
Public	111 (74.0)	39 (26.0)	*p=0.002
**Perceived satisfaction with quality of drugs used in clients management**			χ^2^=10.217
Private	86 (57.3	64 (42.7)	df=1
Public	26 (17.3)	124 (82.7)	*p=0.001
**Satisfaction based on facility utilized by enrollees**			χ^2^=4.835
Private	90 (60.0)	60 (40.0)	df=1
Public	40 (26.7)	110 (73.3)	*p=0.028

* Statistically significant + Likelihood ratio

**Determinants of clients´ satisfaction with quality of care**: at multivariate level, the relationship between selected socio-demographic characteristics, waiting time, facility types utilized by enrollees and level of satisfaction were described using logistic regression. In comparison with NHIS respondents within the age range of 21- 40 years in both the private and public health facilities, clients aged 41-60 years were 2.08 times (95%CI=0.31-3.45, p=0.015) and those above 60 years were 3.26 times (95%CI=0.75-16.16, p=0.074) more likely to be satisfied with the quality of services received under the scheme respectively. Respondents with primary and secondary level of education were 4.37(95%CI = 0.407-46.83, p=0.167) and 4.81 times (95%CI=0.961-24.07, p=0.927) likely to report higher level of satisfaction compared with others respectively. Study participants who were civil servants were 62% less likely to report satisfaction with quality of care received in the two types of health facilities compared with others (OR=0.38, 95%C.I=0.13-1.05, p=0.061). Private health facilities enrollees had significantly lesser odds (73%) of satisfaction with the perceived quality of drugs received in the pharmacies (OR=0.27, 95%C.I=0.39-4.62, p=0.041). Respondents´ who experienced long waiting time were 76% less likely to have higher level of satisfaction compared with those with short waiting time experiences (OR=0.24, 95%C.I=2.394 -7.081, p=0.000) (Table 5).

**Table 5:** relationship between factors associated with clients´ satisfaction and level of satisfaction with quality of care using logistic regression

Variables	Level of satisfaction		Odd	Confidence	P-value
	Low	High	ratio	Interval	
**Age groups (in years)**					
21-40(R)					
41 - 60	90 (55.6)	72 (44.4)	2.08	0.31 to 3.45	*0.015
> 60	50 (36.2)	88 (63.8)	3.26	0.75 to 16.16	0.074
**Level of education**					
Primary	11(28.9)	27 (71.1)	4.37	0.407 to 46.83	0.167
Secondary	117(48.5)	124(51.5)	4.81	0.961 to 24.07	0.927
Tertiary	9 (64.3)	5 (35.7)	3.59	0.83 to 15.57	0.484
Others (R)					
**Occupation**					
Civil servants	113(51.8)	105(48.2)	0.38	0.13 to 1.05	.061
Private sector employee	18 (43.9)	23 (56.1)	0.416	0.67 to 2.84	0.351
Artisan	2 (14.3)	12 (85.7)	4.25	0.59 to 30.89	*0.038
Unskilled workers (R)					
**Perceived quality of drugs given to clients in the pharmacy**					
Public(R)	44(27.1)	118(72.9)			
Private	96(69.6)	42(30.4)	0.27	0.39 to 4.62	*0.041
**Waiting time at facilities prior doctors attention**					
Short(< 1 hour)(R)	60(32.4)	125(67.6)			
Long(≥1 hour)	79(68.7))	36(31.3)	0.24	2.394 to 7.081	*0.000
**Satisfaction based on facility utilized by enrollees**					
Private (R)	79 (52.7)	71(47.3)			
Public	60 (40.0)	90 (60.0)	1.23	0.723 to 2.099	0.443

R-Reference value *Statistical significance

## Discussion

This study is an attempt made by the researchers to assess the determinants of satisfaction with quality of care received by enrollees under the National health insurance scheme in public and private health facilities. The determinants of clients´ satisfaction with quality of care in both private and public health facilities in this study were mainly socio-demographic characteristics, the waiting time experience and the type of facility where services were accessed. The results also revealed a higher overall satisfaction level with quality of care among enrollees using public health facilities compared to those using private health facilities. The study found that satisfaction of clients with the doctors´ care, the waiting time before receiving care was significantly different between the respondents attending public facilities compared with private facilities enrollees. Three most common factors resulting in long waiting time among NHIS clients in public hospitals, were high patient load, few doctors, and record clerks´ verification of client status. Generally, studies have shown that the degree to which health consumers are satisfied with care received is strongly related to the quality of their waiting experience [19-21]. Similarly, the present study showed significant association between clients´ waiting time and their satisfaction. The odds of being more satisfied with services was lower for clients who waited for more than one hour as compared to those who waited less than an hour. When taking into consideration, the findings that more than two-thirds of clients had experienced long waiting time in public health facilities, this may suggest that the facilities had too few health care providers relative to the number of clients. Therefore, exploring the opportunities of employing more health care providers and staggered appointments for enrollees accessing care at public health facilities may be suitable options to reduce the waiting time.

Perceived poor quality of prescribed drugs as reported by more than half (57.3%) of respondents in the private health facilities accounted for the main reason among those who reported low satisfaction with prescribed drugs. Availability of quality drugs has been a challenging issue in many healthcare facilities [22]. A study in Enugu, Southeast- Nigeria ranked non-availability and poor quality of prescribed drugs as one of the leading factors hindering effective utilization of NHIS services [23]. Studies have also reported non-availability of prescribed drugs to be the major complaint associated with lower satisfaction, and access to quality drugs one of the most suggested priorities for improvement of National health insurance scheme [9,24,25]. The NHIS operational guidelines state that “health facilities shall stock generic drugs based on the NHIS drugs list [5,26,27].” It is imperative therefore for the NHIS and HMOs to enforce compliance with the guidelines, while also working with other relevant stakeholders such as National agency for food and drugs administration and control (NAFDAC) to ensure drug quality. Health facilities should also ensure the availability of quality drugs and use identified alternatives that can substitute for the unavailable drug should such occur. Regression analysis of relationship between socio-demographic characteristics and level of satisfaction of NHIS clients in private and public health facilities revealed a statistical significance difference in the age, level of education, occupation, and facility type used by the respondents. This study found a direct relationship between enrollee´s satisfaction and age. Respondents aged 41-60 years were significantly more likely to be satisfied with the quality of care received under NHIS compared with other age-groups. Probable reason for this result, may be due to the fact that older clients were less critical about health care services rendered to them and the Nigerian cultural values which often accords special privileges to elderly clients, is common occurrence even in health care settings. Therefore, the socio-demographic characteristics of clients particularly their age and their perception of quality of care play a major role in people´s decision making process especially in service utilization under the scheme. These findings corroborate the general trend observed in previous satisfaction studies that reported that older clients were more satisfied with service provision than the younger clients [10,28].

Our study also found that clients´ satisfaction with quality of care, in both private and public health facilities were determined by their level of education and occupation. This result is consistent with that reported by a previous study conducted in Sokoto, North-west Nigeria, that also identified marital status, occupation and educational level amongst others as determinants of satisfaction among NHIS enrollees in a public health care facility [11]. Other studies also found that the more educated people are, the more likely, they tend to be critical, while the less educated people are, the more likely they tend to be satisfied [29,30]. Our finding suggests that higher educational level and occupation of the clients could result in higher demands for quality service delivery and consequently cause an increase in the gaps between expectation and services received under the scheme in both facility types. A higher overall level of satisfaction was reported among two-thirds of clients attending the public health facilities than those using private health facilities. This result is however at the lower end of outcomes previously reported in a similar study in Ibadan, Southwestern, Nigeria, which found that 96.4% of clients of public health facilities were more satisfied with the health services they received than 82.4% of clients using private health facilities [8]. Inconsistent findings were reported by a study conducted in Ghana, in which half (50%) of the NHIS patients who sought care at private hospitals or clinics rated their overall satisfaction as “very good” compared to 35% and 30% of those who attended regional/district hospitals and public health centers respectively [25]. Our finding was surprising, because private health facilities were expected to fill the gaps where public health facilities services were inadequate under the National health insurance scheme. Therefore, it is essential to address this, as dissatisfaction could negatively influence patients´ health seeking behaviour, discourage utilization and result in poor enrollment and retention rates; with the attendant adverse effects on the attainment of the NHIS goals and objectives.

Strength: this study gave some insight into the magnitude of NHIS clients´ satisfaction in both private and public health facilities several years after the National health insurance scheme became fully operational. Data was collected from all categories of clients; those attending out-patient clinics, in-patients, and in emergency unit of the private and public health facilities. This is to ensure that all respondents utilized the same general, sensitive, and supporting services at the wards, medical records, pharmacy, and laboratory units of the hospital. This study, therefore, provides useful baseline information for comparative purposes.

Study limitation: a major limitation of this study is that people who may have been dissatisfied with the NHIS services and subsequently made no further use of either the public or private healthcare facilities, may have been left out, which might lead to an underestimation of dissatisfaction levels. Secondly, the questionnaire was interviewer-administered and this might have influenced the responses from the study participants. However, the pretesting of the questionnaire internally did not reveal this limitation. In addition, the research assistants were well-trained to minimize reporting bias and the respondents were assured of confidentiality prior to the conduct of the interview.

## Conclusion

From the findings of the study, clients attending public health facilities were more satisfied with care received under NHIS, compared with those using private health facilities. Client satisfaction with the quality of care under the National health insurance scheme was determined by certain socio-demographic characteristics and factors, which encompass perceived quality of drugs and long waiting time experience. Based on these findings, NHIS policy makers should incorporate the socio-demographic context of clients into the strategic plan of operations of the scheme. Quality improvement strategies should also focus on shortening waiting times through specified appointments and provision of quality of drugs in the public and private health facilities respectively. This will ultimately improve retention of clients under the National health insurance scheme and quality of health service delivery. **Generalizability**: the findings of the study could be generalized to the whole country because the study population in the private and public health facilities consisted of heterogeneous individuals with different socio-economic and cultural backgrounds, which prevailed across Nigeria. However, the dynamic nature of certain regions of Nigeria might exhibit some socio-demographic differences due to cultural, educational, religious and economic status which will require careful consideration.

### What is known about this topic

- *Most of the previous studies assessed the quality of care received by enrollees using clients´ satisfaction as an indicator in either private or public health care facilities;*
- *Evaluating feedbacks from patients about satisfaction with the quality of services accessed, has been documented as an important process in identifying service gaps in previous studies;*
- *There is an increase in the number of insured persons switching from private health care providers to public health care providers and vice versa as a result of poor satisfaction with quality of care received*.

### What this study adds

- *Determinants of satisfaction with quality of care received were mainly the socio-demographic characteristics of NHIS enrollees in public and private health facilities;*
- *NHIS clients receiving care in public health facilities had higher overall satisfaction level(60%) compared with their counterparts receiving care in private health facilities whose satisfaction level was 40%;*
- *Low satisfaction levels were recorded with quality of drugs and long waiting time among the private and public health facilities enrollees respectively*.
